# Supplementary material for: MetaRibo-Seq measures translation in microbiomes
Source: Nat Commun. 2020 Jun 29;11:3268. doi: 10.1038/s41467-020-17081-z (PMC7324362; doi:10.1038/s41467-020-17081-z)
Supplement: Supplementary file 10 — Supplementary Data 7 [file 41467_2020_17081_MOESM10_ESM.zip › File2/Confidence_High_Taxonomy/133012_out.krona.html]

Javascript must be enabled to view this page.

members
magnitude
magnitudeUnassigned
count
unassigned
taxon
rank

133012\_out

70

70
superkingdom
2

1239
phylum
70

186801
class
70

order

SRS054352\_contig\_number\_contig-100\_25862.64158SRS077194\_contig\_number\_contig-100\_2815.190951
2
186802
70

186806
family
2

2
genus
1730

2
species
1897003

SRS050974\_contig\_number\_contig-100\_20970.20971SRS147272\_contig\_number\_contig-100\_11853.11854

66
family
186803

genus
841
1

166486
species

SRS049900\_contig\_number\_9874
1

genus
572511
1

species
33039

SRS048981\_contig\_number\_8507
1


SRS012849\_contig\_number\_22482SRS012969\_contig\_number\_12647SRS014313\_contig\_number\_5646SRS014683\_contig\_number\_contig-100\_140.119768SRS014855\_contig\_number\_contig-100\_3621.95927SRS014948\_contig\_number\_16789SRS015217\_contig\_number\_contig-100\_1088.182692SRS015578\_contig\_number\_34734SRS015854\_contig\_number\_13445SRS016495\_contig\_number\_contig-100\_185.118294SRS017191\_contig\_number\_20086SRS017307\_contig\_number\_contig-100\_35.143890SRS018623\_contig\_number\_contig-100\_2390.127891SRS019030\_contig\_number\_19408SRS019267\_contig\_number\_contig-100\_3489.48773SRS019381\_contig\_number\_contig-100\_533.41919SRS019601\_contig\_number\_31086SRS021484\_contig\_number\_31931SRS022137\_contig\_number\_10398SRS023346\_contig\_number\_8097SRS023526\_contig\_number\_4367SRS042284\_contig\_number\_4656SRS043001\_contig\_number\_9434SRS043701\_contig\_number\_11713SRS047433\_contig\_number\_16764SRS048060\_contig\_number\_13543SRS048870\_contig\_number\_23020SRS049712\_contig\_number\_17850SRS050422\_contig\_number\_contig-100\_214.187181SRS050941\_contig\_number\_17881SRS050998\_contig\_number\_contig-100\_9100.88879SRS053214\_contig\_number\_3988SRS053398\_contig\_number\_24249SRS055982\_contig\_number\_11808SRS058723\_contig\_number\_59SRS063190\_contig\_number\_contig-100\_1044.91070SRS064973\_contig\_number\_contig-100\_5170.5170SRS074586\_contig\_number\_4772SRS075021\_contig\_number\_8674SRS075716\_contig\_number\_contig-100\_3008.3009SRS075773\_contig\_number\_28153SRS076804\_contig\_number\_8690SRS077335\_contig\_number\_2092SRS097920\_contig\_number\_21680SRS103987\_contig\_number\_30931SRS1041036\_contig\_number\_contig-100\_2138.36811SRS1041090\_contig\_number\_2192SRS1041091\_contig\_number\_20890SRS1041133\_contig\_number\_24499SRS1041139\_contig\_number\_contig-100\_2668.25274SRS1041144\_contig\_number\_2424SRS1041146\_contig\_number\_contig-100\_762.763SRS104311\_contig\_number\_18123SRS105153\_contig\_number\_39975SRS1055049\_contig\_number\_16766SRS1055067\_contig\_number\_12688SRS143342\_contig\_number\_10025SRS144362\_contig\_number\_contig-100\_1381.245382SRS144603\_contig\_number\_contig-100\_26645.26646SRS147425\_contig\_number\_10940SRS147614\_contig\_number\_7913SRS893259\_contig\_number\_contig-100\_2713.52637SRS893295\_contig\_number\_3700SRS893327\_contig\_number\_11454
species
39491
64
